# Capacitive Field-Effect EIS Chemical Sensors and Biosensors: A Status Report

**DOI:** 10.3390/s20195639

**Published:** 2020-10-02

**Authors:** Arshak Poghossian, Michael J. Schöning

**Affiliations:** 1 MicroNanoBio, Liebigstr. 4, 40479 Düsseldorf, Germany; 2 Institute of Nano- and Biotechnologies (INB), FH Aachen, Campus Jülich, Heinrich-Mußmannstr. 1, 52428 Jülich, Germany

**Keywords:** chemical sensor, biosensor, field effect, capacitive EIS sensor, pH sensor, enzyme biosensor, label-free detection, charged molecules, DNA biosensor, protein detection

## Abstract

Electrolyte-insulator-semiconductor (EIS) field-effect sensors belong to a new generation of electronic chips for biochemical sensing, enabling a direct electronic readout. The review gives an overview on recent advances and current trends in the research and development of chemical sensors and biosensors based on the capacitive field-effect EIS structure—the simplest field-effect device, which represents a biochemically sensitive capacitor. Fundamental concepts, physicochemical phenomena underlying the transduction mechanism and application of capacitive EIS sensors for the detection of pH, ion concentrations, and enzymatic reactions, as well as the label-free detection of charged molecules (nucleic acids, proteins, and polyelectrolytes) and nanoparticles, are presented and discussed.

## 1. Introduction

Research in the field of biochemical sensors is one of the most fascinating and multidisciplinary topics and has enormously increased over recent years. The global market for biosensor devices grows rapidly and is expected to reach $20 billion by the year 2020 [1]. Due to the small size and weight, fast response time, label-free operation, possibility of real-time and multiplexed measurements, and compatibility with micro- and nanofabrication technologies with the future prospect of a large-scale production at relatively low cost, semiconductor field-effect devices (FEDs) based on an electrolyte-insulator-semiconductor (EIS) system are one of the most exciting approaches for chemical and biological sensing. Ion-sensitive field-effect transistors (ISFET) [2,3,4,5], extended-gate ISFETs [6], capacitive EIS sensors [7,8,9], light-addressable potentiometric sensors [10,11,12,13], silicon nanowire FETs (SiNW-FET) [14,15,16,17], graphene-based FETs [18,19], and carbon nanotube-based FETs [18,20] constitute typical examples of transducer structures for chemically/biologically sensitive FEDs. At present, numerous FEDs modified with respective recognition elements have been developed for the detection of pH, ion concentrations, substrate–enzyme reactions, nucleic acid hybridizations, and antigen–antibody affinity reactions, just to name a few. Moreover, the possibility of an on-chip integration of FED arrays with microfluidics make them very attractive for the creation of miniaturized analytical systems, such as lab-on-a-chip or electronic tongue devices. The possible fields of the application of FED-based chemical sensors and biosensors reach from point-of-care medicine, biotechnology, and environmental monitoring over food and drug safety up to defense and homeland security purposes.

The simplest FED is the capacitive EIS structure, which represents a biochemically sensitive capacitor. In contrast to conventional ISFETs or silicon nanowire FETs, capacitive EIS sensors are simple in layout, easy, and cost-effective in fabrication (typically, without photolithographic or encapsulation process steps). The present status report overviews recent advances and current trends in the research and development of chemical sensors and biosensors based on capacitive field-effect EIS structures. The fundamental concepts, functioning principle, and application of EIS sensors for the detection of pH, ion concentrations, and substrate–enzyme reactions, as well as the label-free detection of charged molecules and nanoparticles, are presented and discussed. The paper also encompasses some key developments of former works. For biochemical sensors based on other types of FEDs, the interested reader is referred to reviews [19,21,22,23,24,25].

## 2. Functioning Principle and Measurement Modes of Capacitive EIS Sensors

**Figure 1:**
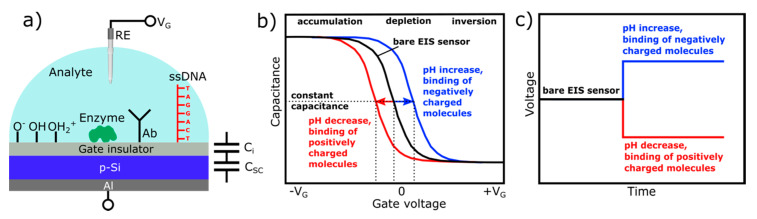
Layer structure of a capacitive electrolyte-insulator-semiconductor (EIS) sensor with different receptor functionalities (pH-/ion-sensing, enzyme, antibody, and DNA) and simplified electrical equivalent circuit (**a**); typical shape of high-frequency capacitance-voltage (*C–V*) curves (**b**); and ConCap response (**c**) of the bare and modified p-type EIS sensor. RE: reference electrode, *V_G_*: gate voltage, Ab: antibody, DNA: deoxyribonucleic acid, *C_i_*: gate-insulator capacitance, *C_SC_*: space-charge capacitance, ssDNA: single-stranded DNA.

Figure 1a schematically shows a typical layer structure of a capacitive EIS sensor and a simplified electrical equivalent circuit. The EIS sensor consists of a semiconductor substrate (in this case, p-type silicon) separated from the solution by a thin (10–100 nm) gate insulator layer (or stack of layers) and a rear-side contact layer (e.g., Al). The gate insulator is assumed to be ideal—that is, no current passes through the insulator. For the operation of EIS sensors, a gate voltage (*V_G_*) is applied between the reference electrode (RE, e.g., conventional Ag/AgCl liquid-junction electrode) and the rear-side contact to regulate the capacitance and set the working point; a small alternating voltage (~10–50 mV) is superimposed to measure the capacitance of the structure. For a proper measurement, the reference electrode should provide a stable potential independent of the pH value of the solution or concentration of the dissolved species.

The electrical equivalent circuit of the EIS sensor is complex and involves components related to the semiconductor, gate insulator, electrolyte/insulator interface, bulk electrolyte, and reference electrode [26,27]. However, for the usual range of a gate insulator thickness and appropriate experimental conditions used (electrolyte solution with ionic strength of >0.1 mM and measurement frequencies of <1 kHz), the equivalent circuit of an EIS sensor can be simplified as a series connection of the gate-insulator capacitance, *C_i_*, and the variable semiconductor space-charge capacitance, *C_sc_* (*V_G_*,*φ*), which is, among others, a function of the gate voltage, *V_G_*, and the electrolyte–insulator interfacial potential, *φ* [27]. Hence, the expression for the total capacitance, *C*, of the bare EIS sensor is given in Equation (1):

(1) $$C=\frac{C_{i}C_{sc}\left(V_{G},\varphi \right)}{C_{i}+C_{sc}\left(V_{G},\varphi \right)}=\frac{C_{i}}{1+C_{i}/C_{sc}\left(V_{G},\varphi \right)}$$

EIS sensors are basically characterized by means of the capacitance-voltage (*C–V*) and/or constant-capacitance (ConCap) mode [28,29]. The typical shape of a high-frequency *C–V* curve for a p-type EIS sensor with characteristic regions of accumulation, depletion, and inversion is exemplarily shown in Figure 1b (black curve); note, an n-type EIS sensor exhibits an identical *C-V* curve; however, the voltage polarity is reversed. If a negative potential (*V_G_* < 0) is applied to the gate, the positively charged holes (majority carriers) will be attracted and accumulated at the semiconductor/insulator interface. In accumulation regime, *C_i_* << *C_sc_*(*V_G_*,*φ*), i.e., the overall capacitance of the EIS structure is determined by the geometrical capacitance of the gate insulator, *C* = *C_i_*, and, thus, corresponds to its maximum capacitance.

When applying a small positive potential (*V_G_* > 0) to the gate, the holes will be pushed away from the interface semiconductor/insulator. As a result, a space-charge region is formed at the semiconductor/insulator interface, which is depleted of mobile carriers (so-called depletion region). The width of the depletion layer is determined by different parameters, such as the applied voltage, doping concentration within the semiconductor, dielectric constant, and insulator thickness. Increasing the amplitude of the applied gate voltage results in an increase of the width of the depletion layer and, consequently, to a decrease of the total capacitance. If the magnitude of the positive gate potential is sufficiently high, the Fermi level bends below the intrinsic level: the concentration of electrons near the semiconductor/insulator interface exceeds the hole concentration, i.e., a thin layer of n-type silicon (so-called inversion layer) is formed, although the substrate is a p-type. By strong inversion, the width of the depletion layer reaches its maximum, and the high-frequency total capacitance of the EIS structure approaches its minimum value.

Equation (1) describes the total capacitance of the EIS sensor without defining the origin of the potential generation at the interface electrolyte/insulator. When fixing the applied gate voltage, *V_G_*, the only variable component is the interfacial potential, *φ*, which is analogous to the effect of applying an additional voltage to the gate. Since FEDs are potential-/charge-sensitive devices, any kind of chemical and/or electrical change at or nearby the interface electrolyte/gate can be detected by the EIS sensor. Those changes can be induced by biochemical reactions, when the capacitive field-effect sensor is functionalized with a particular chemical and/or biological recognition element, such as a pH-sensitive layer, ionophore, enzyme, antibody, nucleic acid, etc. For (bio-)chemical sensor applications and for investigating charge effects in such capacitive EIS structures, the shift of the *C*–*V* curves along the voltage axis (Δ*V_G_*) in the depletion region (Figure 1b) is more important. The direction of these potential shifts depends on the charge sign of the adsorbed chemical and/or biological species. For example, in case of a p-type EIS structure, an increase of the analyte’s pH value or binding of the negatively charged species to the gate surface will decrease the width of the depletion layer, yielding an increase of the depletion capacitance in the Si. By this, the total capacitance of the sensor will increase, and the *C*–*V* curve will shift to the direction of more positive (or less negative) gate voltages (Figure 1b, blue curve). Conversely, a pH decrease or the electrostatic adsorption or binding of positively charged species to the gate surface will lead to an increase of the width of the depletion layer; the space-charge capacitance will decrease. As a consequence, the total capacitance of the EIS sensor will also decrease, resulting in a shift of the *C*–*V* curve towards more negative (or less positive) gate voltages (Figure 1b, red curve).

The amplitude, as well as the direction of potential shifts, can directly be determined from dynamic ConCap-mode measurements (see Figure 1c). In addition, the ConCap mode enables real-time monitoring of the sensor signal and investigation of the response time, drift, and hysteresis of the EIS sensor. In the ConCap mode, the total capacitance of the EIS sensor at the working point is kept constant by using a feedback circuit, which applies an instantly sign-inverted voltage to the EIS sensor. Usually, the working point for the ConCap mode is set within the linear range of the depletion region of the *C*–*V* curve.

In the following sections, an origin of different mechanisms of interfacial potential generation (e.g., pH and ion-concentration changes, enzymatic reactions, adsorption, and binding of charged molecules and nanoparticles) is described, which enables EIS devices to be sensitive to numerous chemical and biological species, as well as to discuss the physicochemical phenomena underlying the transduction mechanism of EIS-based chemical sensors and biosensors.

## 3. Chemical Sensors and Biosensors Based on Capacitive EIS Structures

### 3.1. EIS pH Sensor

Field-effect pH sensors based on an EIS system detect potential (charge) changes at the electrolyte/gate-insulator interface, resulting from the changes in the local or bulk pH. It is known that the gate-insulator material in the first ISFET was SiO_2_, which is not the best pH-sensitive material, having a low sensitivity, a narrow linear pH range, a relatively high drift, and a large hysteresis (see, e.g., [9,30,31]). Therefore, other oxides, like Al_2_O_3_ [32,33,34], Ta_2_O_5_ [29,35,36], ZrO_2_ [37], HfO_2_ [38,39,40,41], CeO_2_ [42], Gd_2_O_3_ [43,44], Ti-doped Gd_2_O_3_ [45], Lu_2_O_3_ [46], Nd_2_O_3_ [47], Yb_2_O_3_ [48], Dy_2_TiO_5_ [49], Er_2_TiO_5_ [50], PbTiO_3_ [51], YTi_x_O_y_ [52], Tm_2_Ti_2_O_7_ [53], and barium strontium titanate (BST) [54,55,56], as well as Si_3_N_4_ [32,57] and nanocrystalline diamond (NCD) [58], have been proven as pH-sensitive gate insulators for EIS sensors. Some of the recent results, including the pH-sensitive material used, deposition technique, pH sensitivity, pH range, drift, and hysteresis, are summarized in Table 1.

In most cases, these pH-sensitive materials are deposited on top of a SiO_2_ layer by means of different deposition techniques (e.g., thermal oxidation, chemical vapor deposition, electron-beam evaporation, sputtering, pulsed laser deposition, atomic layer deposition, and sol-gel technique), thus forming a stacked gate insulator (e.g., SiO_2_–Si_3_N_4_, SiO_2_–Al_2_O_3_, or SiO_2_–Ta_2_O_5_). The upper layer of the double-insulator structure typically serves as the pH-sensitive material, whereas the SiO_2_ layer provides a stable Si-SiO_2_ interface with a low density of states.

The main parameters, which determine the analytical characteristics of the EIS pH sensors, are sensitivity, selectivity, stability (or drift), linear pH range, hysteresis, and response time. Other important parameters include the temperature stability, light insensitivity, reproducibility, and lifetime. These characteristics are most thoroughly studied for Si_3_N_4_, Al_2_O_3_, and Ta_2_O_5_ layers, which belong to the best pH-sensitive materials. At present, Si_3_N_4_, Al_2_O_3_, and Ta_2_O_5_ serve as pH-sensitive gate insulator materials in commercial pH-ISFETs available from many companies producing electrochemical sensors. Other more exotic pH-sensitive materials described in Table 1 sometimes show nearly Nernstian sensitivity but have been only rarely studied.

At present, the generally accepted and successfully applied model describing the functional mechanism of pH-sensitive FEDs with inorganic gate insulators (e.g., oxides and nitrides) is the so-called site-binding model, which was originally developed to describe the charging mechanism of oxide surfaces immersed in solution [59]. Due to the hydration, the surface of oxides contains neutral amphoteric hydroxyl groups in the particular cases of Ta_2_O_5_ and TaOH groups. Dependent on the pH value of the solution, these surface sites are either able to bind or release a proton (H^+^), resulting in protonated (TaOH_2_^+^) or deprotonated (TaO^−^) groups according to the following reactions in Equations (2) and (3):

TaOH ⇄ TaO^−^ + H^+^ (2)

TaOH_2_^+^ ⇄ TaOH + H^+^ (3)

At a pH range of pH > pH_pzc_, the oxide surface of the capacitive field-effect sensor will be negatively charged, whereas at a pH regime with pH < pH_pzc_, it is positively charged; the value pH_pzc_ is defined as the pH value at the point of zero charge. Consequently, the pH-dependent surface charge of the gate insulator will modulate the space-charge capacitance in the Si and, finally, the overall capacitance of the capacitive field-effect structure. Note that, in contrast to oxides, the pH sensitivity of Si_3_N_4_ can be explained by utilizing the modified site-binding theory. This theory considers the presence of two different types of surface sites: SiOH (silanol) and SiNH (amine) groups [60].

Commonly, the pH sensitivity of capacitive field-effect sensors is determined as the potential change (*φ*) at the interface electrolyte/gate-insulator, referred to as the change in the bulk pH; see Equations (4) and (5) [61]:

(4) $$\frac{\delta \varphi }{\delta pH}=-2.3\frac{kT}{q}\alpha ,\;\mathrm{with}$$

(5) $$\alpha =\;\frac{1}{\left(2.3\;kTC_{diff}/q^{2}\beta_{int}\right)+1}$$

In the equations, *α* represents a dimensionless sensitivity parameter that varies between 0 and 1, *β_int_* is the surface intrinsic buffer capacity, which characterizes the ability of the oxide surface to release or bind protons, *C_dif_* is the differential double-layer capacitance (depending on the ion concentration of the solution), *k* is the Boltzmann’s constant, *T* is the temperature, and *q* is the elementary charge (1.6 × 10^−19^ C).

From Equations (4) and (5), the maximum Nernstian sensitivity (59.3 mV/pH at 25 °C) can be obtained only if *α* ≈ 1, i.e., in the case of a large value of the surface-buffer capacity (high density of surface-active sites) and a low value of the double-layer capacitance (low electrolyte concentration). For materials with *α* < 1, a sub-Nernstian response can be expected. Thus, oxides with a high density of the surface sites (e.g., Ta_2_O_5_ with ~10^15^ sites/cm^2^ or Al_2_O_3_ with 8 × 10^14^ sites/cm^2^ [61]) possess a high pH sensitivity, whereas for SiO_2_ with less surface sites (5 × 10^14^ sites/cm^2^ [61]), a low pH sensitivity could be expected that, in fact, was observed in experiments.

Determination of the pH value is one of the most important measurements in many fields, including clinical diagnostics, biotechnology, food, pharmaceutical and cosmetic industries, agriculture, environmental monitoring, water purification, etc. Currently, the most often used electrode is a traditional pH glass membrane electrode. However, glass electrodes have two main problems, namely the fragility of the glass membrane and easy fouling in aggressive media.

In comparison to pH glass electrodes, FEDs are often advertised due to their resistance to breakage (unbreakable pH sensor). Therefore, breakable pH glass electrodes are gradually replaced by nonglass, unbreakable pH-ISFETs in many in-line process-monitoring systems [62]. Besides being unbreakable, often, additionally, those sensors must be CIP-(cleaning-in-place) or SIP-(sterilization-in-place) feasible. According to [62], commercially available pH-ISFETs have a limited lifetime by CIP procedures, which use highly caustic media and high temperatures to clean process vessels. Therefore, Schöning et al. studied the CIP suitability of pH-sensitive Ta_2_O_5_ EIS sensors, where the Ta_2_O_5_ films were fabricated by thermal oxidation of a Ta layer [29]. For these sensors, even after running 30 CIP cycles, a nearly-Nernstian sensitivity of 57 ± 1.5 mV/pH was recorded. Note, each CIP cycle included cleaning procedures in a 4% NaOH solution at 80 °C during 15 min and, subsequently, in 0.65% HNO_3_ solution at 80 °C during 5 min. No visible degradation of the Ta_2_O_5_ films of the EIS sensors was observed. These experiments demonstrated that, in addition to the high pH-sensitive behavior, Ta_2_O_5_ films have also high corrosion-resistant properties. Such sensors can be placed in direct contact with food for pH measurements without the risk of broken glass fragments. In further experiments, the authors also demonstrated the suitability of Ta_2_O_5_-gate EIS structures for SIP procedures [63]. These sensors were integrated into a lab-scale bioreactor and successfully tested for continuous pH monitoring during cell-culture fermentation processes [64]. In addition, Ta_2_O_5_-gate EIS pH sensors were applied for measuring the extracellular acidification rate of *Escherichia coli* upon glucose pulses [65]. Further examples are EIS sensors with BST films as pH-sensitive material for the pH control in a biogas digestate [56] or an estimation of the acid content in rancid butter samples with a Si_3_N_4_-gate EIS pH sensor [66]. Finally, EIS pH sensors with Ta_2_O_5_ films prepared by radio frequency magnetron sputtering were utilized for the real-time quantitative detection of DNA (deoxyribonucleic acid) amplification via loop-mediated isothermal amplification [36]. Here, during the elongation reaction, protons are produced (proportional to the number of nucleotides incorporated), thus resulting in a pH shift of the surrounding solution that is detected by the EIS pH sensor.

### 3.2. Ion-Sensitive EIS Sensors

EIS sensors selective towards ions other than protons can be obtained by modification of the original gate insulator—for instance, with additional organic or inorganic ion-sensitive membranes, suitable recognition molecules, or ion implantation. The easiest and most convenient method is deposition (e.g., via spin-, dip-, or drop-coating techniques) of an ion-selective polymeric membrane containing a respective ionophore atop the gate-insulator surface. Since the ion-sensitive membrane is permeable for ions, generally, the ion-exchange process is responsible for the potential generation at the electrolyte/membrane interface (given by the well-known Nernst or modified Nernst-Nikolsky equation), which is detected by the EIS sensor. Principally, any membrane composition implemented in conventional ion-selective electrodes can also be used in EIS sensors. Poly(vinyl chloride) is one of the most commonly applied matrices for ionophore-containing membranes. For example, a K^+^-sensitive EIS sensor consisting of a p–Si–SiO_2_–Si_3_N_4_ structure covered with a valinomycin (ionophore)-containing poly(vinyl chloride) membrane was realized in [67]. Before membrane deposition, the Si_3_N_4_ surface was silanized in order to suppress the intrinsic pH sensitivity of the Si_3_N_4_ layer and to reduce the influence of the pH of the solution on the K^+^-sensitive response, as well as to improve the membrane adhesion. Sensors exhibited a K^+^-sensitivity of 53 ± 2 mV/pK in a linear concentration range of 10^−5^–10^−1^ M, a lower detection limit of 5 × 10^−6^ M, a small hysteresis of ~2 mV, and a fast response time of 5–12 s. The developed sensors were capable for K^+^-ion concentration measurements for at least six months. In a further work, the authors studied the impact of the membrane impedance on the characteristics of the K^+^-sensitive EIS sensor [68]. It was shown that a high series resistance of the ion-sensitive membrane can lead to a frequency-dependent distortion of the *C–V* curves, especially by measurements at high frequencies. In [69], an EIS calcium sensor was developed for the determination of the risk of urinary stone formation. In this application, an Al–p–Si–SiO_2_–Ta_2_O_5_ structure was utilized that was modified with an ion-selective poly(vinyl chloride) membrane with the Ca^2+^ ionophore ETH 1001. The EIS calcium sensor had a Ca^2+^ sensitivity of 27 ± 2 mV/pCa in the concentration range of 0.1–10 mM and a lower detection limit of 0.01-mM Ca^2+^; its response time was about ~30 s. Measurements in real test samples have been done for the determination of the Ca^2+^ concentration in native urine. Another membrane (siloprene) containing trioctylphosphine oxide as the ionophore deposited on a Si–SiO_2_–Si_3_N_4_ structure was utilized to realize an EIS sensor sensitive towards hexavalent chromium (CrVI) [70]. A good selectivity, a quasi-Nernstian sensitivity of 27.6 mV/pCr in the concentration range of 10^−4^–10^−1^ M Cr(VI), and a detection limit of 10^−5^ M Cr(VI) was reported. More recently, a perchlorate anion (ClO_4_^−^)-sensitive EIS sensor was developed by modification of the HfO_2_ gate surface with a Co(II) phthalocyanine acrylate polymer (Co(II)Pc-AP) [71].

The main problems of ion-sensitive EIS sensors based on polymeric membranes are the poor adhesion of the membrane to the gate surface and possible ionophore leakage, thus limiting the sensor lifetime. These problems can be overcome by using ion-sensitive inorganic materials, which can be deposited onto the gate surface by thin-film deposition techniques, being compatible with semiconductor technology and, therefore, with the EIS fabrication. For example, a fluoride (F^−^)-selective EIS sensor was developed by the thermal deposition of polycrystalline lanthanum fluoride (LaF_3_) films on the SiO_2_ gate surface [72]. The sensor exhibited a high sensitivity of 52.3 mV/pF in the F^−^-ion concentration range of 10^−2^–10^−6^ M, a relatively low hysteresis (5.1 mV), and a small drift (0.67 mV/h).

Due to the low solubility in water and the possibility of deposition and patterning of thin-film membranes with various compositions by microelectronic techniques, chalcogenide-glass films are very promising for the development of EIS sensors sensitive towards various heavy metal ions. For example, EIS sensors with thin-film chalcogenide-glass membranes of CdSAgIAs_2_S_3_ and PbSAgIAs_2_S_3_ were developed for the detection of Pb^2+^- and Cd^2+^ ions, respectively [73], which belong to the most toxic species of superficial and ground waters. The chalcogenide-glass films were prepared onto the Ta_2_O_5_ gate surface by means of a pulsed laser deposition technique that enables the stoichiometric transfer of these multicomponent materials from the original target to the sensor surface. The sensitivity to Pb^2+^- and Cd^2+^ ions was 24 mV/pPb and 23 mV/pCd, respectively, in a linear concentration range of about 5 × 10^−6^–10^−2^ M. A detection limit of ~3 × 10^−6^ M and response time of 1 min was reported for both sensors.

A completely other approach to make the gate surface ion-sensitive is based on its modification via ion implantation—a technique also compatible with silicon technology. Moreover, multiple membranes sensitive to various ions can be fabricated on the wafer level using multiple implants and different photolithographic masks. The ion-implantation technique was applied to develop Na^+-^- and K^+^-sensitive EIS sensors by implanting Na^+^ or K^+^ ions into the oxidized silicon nitride through an Al buffer layer [74,75]. Although, these sensors demonstrate good sensitivity (52 mV/pNa and 49 mV/pK [75]), the response to interfering ions and pH was non-negligible, evidencing a poor selectivity of implanted ion-sensitive layers. Finally, several types of EIS sensors sensitive towards Ni^2+^ [76], Cu^2+^ [77], and Hg^2+^ [78] were realized via the deposition of ion-recognition molecules (macrocyclic compounds such as calixarenes [79]) directly onto the gate surface.

### 3.3. Enzyme-Modified EIS Biosensors

Due to their catalytic activity, enzymes are frequently used as bioreceptors. Enzyme-modified capacitive EIS (EnEIS) biosensors are typically constructed via the immobilization of enzymes onto the gate surface. In general, the operation principle of an EnEIS biosensor can be explained as follows: During the enzymatic reaction of the enzyme with its substrate, either reactants are consumed or products are generated; this concentration change is detected by the EIS sensor. A vast majority of EnEIS biosensors are built up of pH-sensitive EIS structures, which detect hydrogen ions produced or consumed during the enzymatic reaction. Equations (6) and (7) present typical examples of enzymatic reactions generating hydrogen ions (pH decrease) and consuming hydrogen ions (pH increase) by using penicillin/penicillinase and urea/urease as the model substrate/enzyme system:

penicillinase penicillin + H_2_O → penicilloic acid + H^+^ (6)

urease NH_2_-CO-NH_2_ (urea) + 2H_2_O + H^+^ → 2NH_4_^+^ + HCO_3_^−^ (7)

For example, in the case of a penicillin-sensitive EnEIS biosensor, the enzyme penicillinase catalyzes the hydrolysis of penicillin to penicilloic acid, yielding an increase of the H^+^-ion concentration near the gate region of the EIS sensor. A resulting local pH change near the surface of the pH-sensitive layer will alter the gate-surface charge (similar to the pH-sensitive EIS sensors described in Section 3.1), which, in turn, will modulate the space-charge capacitance in the Si and, consequently, the total capacitance of the EnEIS biosensor. Hence, the amplitude of the output signal of the biosensor will be determined by the concentration of penicillin in the sample solution. Owing to the described working principle, a large sensor signal and high analyte sensitivity can be expected for EnEIS structures with gate-insulator materials exhibiting a high pH sensitivity, as well as by measurements in low buffer capacity solutions.

The choice of the appropriate enzyme immobilization strategy is essential for the development of enzyme biosensors with good performance (high sensitivity, selectivity, operational and storage stability, and fast response time) and represents one of the most critical points for the construction of EnEIS biosensors. Therefore, a broad spectrum of enzyme immobilization methods, such as physical adsorption, the layer-by-layer (LbL) technique, covalent binding, crosslinking, affinity coupling, entrapment within a polymeric membrane or hydrogel beads, etc., has been utilized. Each of these immobilization strategies has its own pros and cons. Common drawbacks are often a low or unreproducible enzyme-surface density, as well as the necessity of complicated surface functionalization or modification procedures.

At present, a large group of EnEIS biosensors by applying various gate materials, enzyme membrane compositions, or immobilization methods have been developed for the detection of different analytes such as acetoin [80], creatinine [81,82,83], cyanide [84], formaldehyde [85], glucose [53,81,82,86,87,88,89,90,91,92,93,94,95], pesticides of paraoxon [96] and atrazine [97], antibiotics of ampicillin, amoxicillin [98], and penicillin [58,98,99,100,101,102,103], triglycerides [66], and urea [82,89,90,91,92,95,104,105,106,107,108,109,110,111]. Some recent developments are summarized in Table 2.

**Figure 2:**
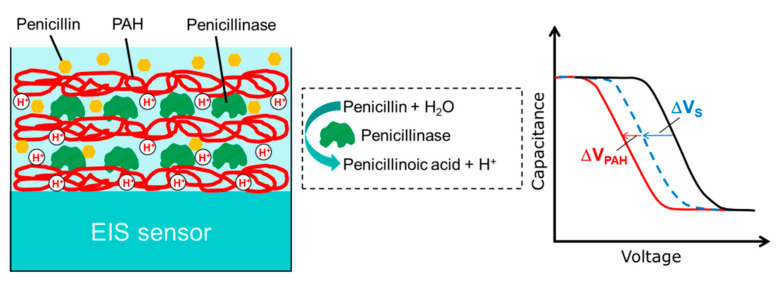
Functioning principle of a penicillin-sensitive EIS biosensor modified with a pH-responsive weak polyelectrolyte (PE)/enzyme multilayer: schematic structure (left), enzymatic reaction of the catalyzed hydrolysis of penicillin by the enzyme penicillinase (middle), and expected shift of the *C*-*V* curves of the EIS sensor (right). Δ*V_S_*: shift of the *C*–*V* curve due to local pH change near the gate surface induced via the enzymatic reaction and Δ*V_PAH_*: additional shift of the *C*–*V* curve induced by charge changes of weak PE macromolecules. Reproduced from Ref. [113] with permission from Springer International Publishing.

During the last years, several new pH-sensitive gate materials and enzyme immobilization strategies have been proposed to improve the working parameters of EnEIS biosensors. Examples are the adsorption of enzymes onto or within a LbL-deposited dendrimer/carbon nanotube (CNT) multilayer [99,106], the functionalization of EIS sensors with gold [86,112] or magnetic nanoparticles [81,88,104] covered with immobilized enzymes, and the encapsulation of enzymes within alginate beads or a gel layer [82,93,105,108]. In addition, a novel strategy for enhanced field-effect biosensing utilizing capacitive EIS devices modified with a pH-responsive weak polyelectrolyte (PE)/enzyme multilayer was proposed in [101]. The EnEIS biosensor responds to both the local pH change near the gate surface induced via the enzymatic reaction and the pH-dependent charge changes of weak PE macromolecules, resulting in a large sensor signal and higher analyte sensitivity (see Figure 2). Moreover, by the incorporation of enzymes within a multilayer, a larger amount of immobilized enzymes per active sensor area, reduced enzyme-leaching effects, and an enhanced biosensor lifetime can be expected.

**Figure 3:**
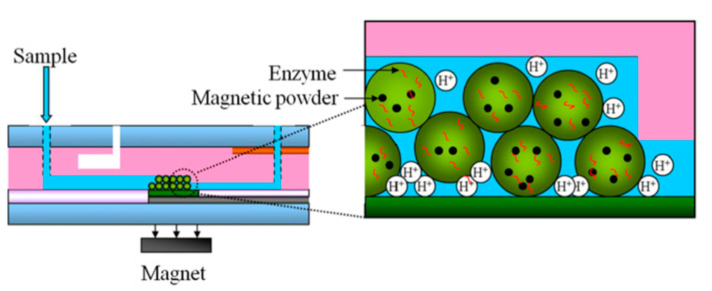
Operating principle of a microfluidic chip. Enzyme-carrying alginate microbeads are immobilized on the EIS-sensor surface by means of an external magnetic field. The sample is injected into the microchannel and reacts with the enzyme contained in alginate beads. The change in potential on the sensor surface induced by the release of hydrogen ions during the reaction process is measured. Reproduced from Ref. [82] with permission of Elsevier.

This generic concept was demonstrated by developing a penicillin-sensitive EnEIS biosensor based on a capacitive p–Si–SiO_2_ structure functionalized with a LbL-prepared poly(allylamine hydrochloride) (PAH)/penicillinase multilayer. The developed penicillin biosensor possesses a high sensitivity of 100 mV/dec in a linear range of 25 µM–10 mM and a low detection limit of 20 µM [101]. The loss of penicillin sensitivity after two months was about 10%. A similar strategy was used in [106] for the development of a urea biosensor based on a Ta_2_O_5_-gate EIS structure modified with a dendrimer/CNT/urease/CNT LbL multilayer. The sensor arrangement with the enzyme urease sandwiched between two CNT layers showed an approximately two-fold higher urea sensitivity in comparison to an arrangement with the enzyme urease immobilized atop of the dendrimer/CNT multilayer. To increase the dynamic range of urea detection (0.1–100 mM), the EIS sensor surface was covered with ferric oxide (Fe_3_O_4_) magnetic nanoparticles modified with a LbL multilayer of PAH/PSS (poly(sodium 4-styrenesulfonate))/PAH/urease [104]. The immobilization of enzymes on magnetic beads was also applied for the development of glucose- and creatinine-sensitive EnEIS biosensors [81]. The enzymes glucose oxidase or creatinine deaminase were covalently immobilized on the magnetic beads, and then, the beads were positioned (via an external magnetic force) on the surface of a Dy_2_TiO_5_-gate EIS sensor integrated within a microfluidic chip. In further works, the alginate microbead-containing magnetic particles were used as enzyme carriers for the creation of EIS-based glucose, creatinine, and urea biosensors (see Figure 3) [82,93].

The alginate microbeads with embedded magnetic particles and the enzymes glucose oxidase or creatinine deaminase or urease were positioned onto the pH-sensitive Dy_2_TiO_5_ gate via an external magnet. The main advantages of this approach are the capability for detection of multiple analytes using the same sensor chip and possibility of replacing alginate microbeads by new beads once the encapsulated enzyme is consumed.

Another concept for a reusable EnEIS glucose biosensor using a disposable hydrogel/enzyme layer was proposed in [53]. Here, the Tm_2_Ti_2_O_7_-gate surface was covered by a thermosensitive poly(N-isopropylacrylamide) hydrogel layer containing the enzyme glucose oxidase. Owing to the phase-tunable characteristics of the hydrogel, the enzyme/hydrogel film was easily loaded onto the surface of the EIS transducer at an increased temperature and then, after measurements, completely removed from the surface by decreasing its temperature, while the EIS sensor underneath was preserved.

**Figure 4:**
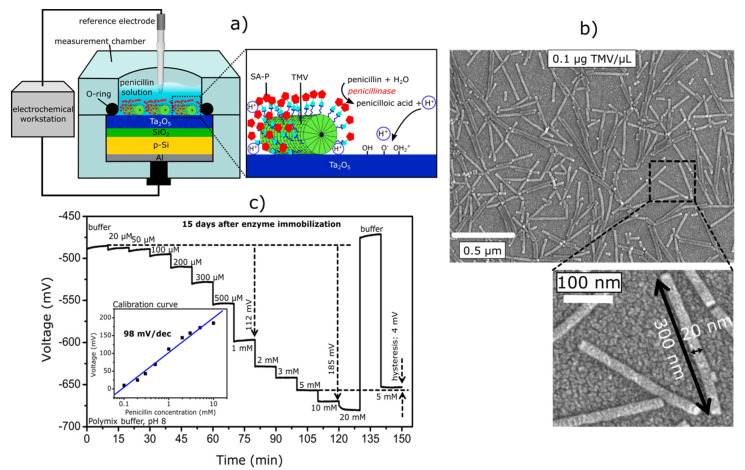
Schematic structure of an enzyme-modified capacitive EIS (EnEIS) biosensor modified with *tobacco mosaic virus* (TMV) particles as enzyme nanocarriers (**a**), scanning electron microscopy image of TMV particles on the Ta_2_O_5_ surface (**b**), and the sensor signal in buffer solution with different penicillin concentrations (**c**). Adapted from Ref. [103] with permission of Elsevier.

More recently, a novel promising approach for the development of EnEIS biosensors was described in [103,114], where a highly sensitive penicillin biosensor with a superior lifetime was realized by means of modification of a Ta_2_O_5_-gate EIS structure with *tobacco mosaic virus* (TMV) particles as scaffolds for the dense immobilization of enzymes. The TMV has a nanotube-like structure with an average length of 300 nm, an outer diameter of 18 nm, and an internal channel of 4 nm in diameter [115,116]. The TMV surface holds thousands of sites capable for the coupling of various biological receptors, including enzymes. The enzyme penicillinase was immobilized onto the biotinylated TMV surface via a bio-affinity binding of commercially available streptavidin–penicillinase conjugates to biotin. Figure 4 shows the schematic structure of the EnEIS biosensor modified with TMV particles as enzyme nanocarriers (a), a scanning electron microscopy image of TMV particles on the Ta_2_O_5_ surface (b), and the sensor signal in buffer solution with different penicillin concentrations (c).

The biosensor had a high penicillin sensitivity of ~92 mV/mM in the linear range of 0.1–10 mM, a low detection limit of 50 μM, and an exceptional long-term stability of at least one year. Most likely, this novel approach may be adapted to other enzymes. The results obtained in [103] demonstrate a great potential for the integration of plant virus/receptor nanohybrids with electronic chips, thereby opening new opportunities in advanced biosensing technologies.

The existing enzyme-layer deposition methods often apply manual techniques (e.g., dip- or drop-coating), which are simple but poorly reproducible and relatively time-consuming. Advanced multisensor array and biochip technologies require the controlled and spatially resolved immobilization of a defined amount of biomolecules on the particular transducer surface. Therefore, a nano-spotter, a device for noncontact ultra-low volume dispensing, has been examined for spatially resolved deposition of the enzyme penicillinase onto the Ta_2_O_5_-gate surface of an EIS structure [102,117]. The nano-spotted penicillin biosensor exhibited identical sensing characteristics (in terms of sensitivity, linear range, and lower and upper detection limit) as the drop-coated EIS sensor counterpart. However, the advantage of nano-spotting is its capability for creating an array of patterned micro-spots immobilized with various enzymes.

An evaluation of the results on EnEIS biosensors reported in the literature and partially included in Table 2 reveals that a direct comparison of their basic working characteristics is difficult because of different gate insulators, enzyme-immobilization methods, enzyme activity, or buffer capacity used. Typical problems of EnEIS biosensors are similar to that of enzyme-modified FETs and include, for instance, the rather narrow linear measurement range, the detection limit, the relatively slow response, and the dependence of the sensor signal on the enzyme-immobilization method, buffer capacity, and pH value of the test sample. There are only a few papers, where the hysteresis effect, operational and storage stability, reproducibility, and lifetime of EnEIS biosensors have been discussed. Although, during the last years, a number of technological solutions have been proposed and tested to solve these problems and to improve the working parameters of EnEIS biosensors, their transfer from research laboratories to real-life applications remains rather slow. Some examples for practical applications include EnEIS biosensors for the detection of glucose [81,82,87], creatinine [81,82], urea [82], and total triglyceride level [66] in human serum, glucose level in whole blood [88], acetoin in diluted white wine samples [80], and penicillin in bovine milk [103].

Another application field of EnEIS biosensors is their use in enzyme-based logic gates that mimic the working principle of electronic logic gates—basic elements of conventional computing. An integration of biomolecular—in particular, enzyme logic principles—with electronic transducers could facilitate novel digital biosensors with a logic output signal in YES/NO format, logically triggered actuators and drug-release devices, and even intelligent closed-loop sense/act/treat systems with enormous potential in advanced point-of-care diagnostics, personalized medicine, and theranostics [118,119,120,121,122,123,124]. The possibility of interfacing of enzyme logic gates with FEDs was first demonstrated in [125], where a capacitive field-effect EIS sensor consisting of an Al–p–Si–SiO_2_ structure modified with pH-responsive gold nanoparticles (AuNP) was applied for designing single **AND** and **OR** logic gates. The operation of EIS-based enzyme logic gates developed in [125] was based on bulk pH changes induced by biochemical reactions activated by different combinations of chemical input signals (substrates). The enzymatic part of the system is responsible for sensing of the chemical signals and their logic treatment. As a result of bulk pH changes, the EIS sensor generates an electronic signal corresponding to the logic output produced by the enzymes.

The first example demonstrating the successful transfer of biomolecular logic principles from the bulk solution to the surface of FEDs was reported in [126], where **AND-Reset** and **OR-Reset** logic gates were realized by immobilizing multi-enzymes onto Ta_2_O_5_-gate EIS structures via entrapment within a polymeric membrane. In contrast to [125], the operation of these enzyme logic gates is based on local pH changes induced by an enzymatic reaction (or cascade of reactions), while the pH value of the bulk solution remains practically unchanged. Thereby, multiple enzyme logic gates, or even logic systems working in the same solution, as well as individual addressing and switching of the respective logic gates, is possible. In further works, other enzyme logic gates such as **AND-Reset** and **OR-Reset** gates with an integrated **Reset** function, **Controlled NOT** (**CNOT**), and **XOR** were developed by immobilization or physical adsorption of multi-enzymes onto Ta_2_O_5_-gate EIS structures [127,128,129], demonstrating the successful interfacing of enzyme logic principles with semiconductor FEDs.

### 3.4. Label-Free Detection of Charged Molecules

The detection of adsorption and binding of charged molecules is of great interest for numerous application fields, ranging from clinical diagnostics, environmental monitoring, genetics and the drug industry over biosensors, DNA-chips, and protein-microarray technology up to the fundamental studies of molecular interactions at the solid/liquid interface. Since, in the majority of cases, biological molecules are difficult to detect via their intrinsic physical properties, biosensors often require the labeling of target analytes with different markers or reagents (e.g., enzymatic, redox, or fluorescent) to facilitate the signal readout. In spite of their high sensitivity, label-based techniques suffer from the fact of being time-consuming and labor- and cost-intensive. For the development of fast, simple, and inexpensive biosensors, label-free technologies, which utilize intrinsic physical properties of the analyte molecule to be detected (e.g., charge, electrical impedance, molecular weight, dielectric permittivity, or refractive index), are more favorable.

Since EIS sensors represent charge-sensitive devices (see discussion in Section 2), they can also detect any kind of charged molecules adsorbed or bound onto their gate surface. This way, the coupling of charged molecules, nanoparticles, and even inorganic/organic nanohybrids onto capacitive field-effect sensors is a very promising strategy to actively tune their electrochemical properties, especially with regards to label-free biosensing. Recent examples towards the label-free, direct electrical detection with the help of capacitive EIS sensors consider various kinds of charged molecules [7,27,130,131,132] and charged nanoobjects (nanoparticles and nanotubes) [112,113,133,134]. In this section, key developments of label-free EIS biosensors will be introduced, which mainly focus on electrostatic DNA detection, the detection of proteins, and oppositely charged PE macromolecules; all of them are monitored by their intrinsic molecular charge. In addition, nanoparticle-modified EIS sensors will be presented.

#### 3.4.1. Detection of DNA Molecules

In recent years, DNA biosensors have been increasingly recognized as powerful tools in many fields of application, including molecular diagnostics, pathogen identification, drug screening, food safety, forensic and parental testing, or detecting biowarfare agents. The vast majority of DNA-modified EIS (DNA-EIS) biosensors reported in the literature is based on detecting a DNA-hybridization reaction [7,27,135,136,137,138], although the detection of single-stranded DNA (ssDNA) [8,132] and double-stranded DNA (dsDNA) [132,139,140,141], as well as other DNA-recognition events, like single-base mismatch [130], the by-product (protons) of the nucleotide base incorporation reaction [36], and DNA amplification by polymerase chain reaction (PCR) [139,140,142,143,144], have been demonstrated as well.

To our knowledge, the first successful experiment on DNA-hybridization detection with an EIS structure using synthetic homo-oligomers as a model system was reported in [145]. During the DNA-hybridization event, the probe ssDNA molecules with known sequences identified their complementary target DNA molecules (cDNA), and a dsDNA helix structure with two complementary strands was formed. The hybridization reaction was highly efficient and specific, even in the presence of noncomplementary nucleic acids. Usually, capacitive DNA-EIS biosensors detect the hybridization event on-chip: First, probe ssDNA molecules (of known sequences) are immobilized onto the gate surface; this is done by, e.g., adsorption [130,132,137] or covalent attachment [27,146]. In the next step, the target cDNA molecules are detected by in-situ real-time monitoring or ex situ. For in-situ real-time monitoring, the sensor signal is directly recorded during the hybridization process [130], whereas, for ex-situ detection, the response of the EIS sensor is compared before and after hybridization [7,132,135,137]. Capacitive EIS sensors detect DNA molecules electrostatically by their intrinsic molecular charge. During the hybridization process, the negatively charged (due to the phosphate-sugar backbone) target cDNA molecules will effectively alter the charge applied to the gate surface of the EIS sensor, which, in turn, will modulate the space-charge distribution in the semiconductor and capacitance of the EIS structure.

One obstacle of all kinds of FEDs—also including DNA-EIS biosensors for electrostatic DNA detection—is the screening of the DNA charge by mobile counter ions in the surrounding solution. Capacitive field-effect sensors are able to detect charge/potential changes occurring directly at the gate surface or within the order of the Debye length from the surface; note, the Debye length is inversely proportional to the ionic strength of the analyte (e.g., in physiological solutions (~150 mM), it amounts to ~0.8 nm [22]). In the case of DNA molecules tethered to the gate surface, the DNA charge is not confined directly to the interface, but it is distributed through some distance away from the surface, which depends on the DNA length. As it was discussed in [147], the effectivity of electrostatic coupling between the DNA charge and gate surface and, thus, the generated DNA-hybridization sensor signal will strongly drop with increased distance between the DNA charge and gate surface. The use of additional long linker molecules for ssDNA immobilization could also result in a smaller hybridization signal. In contrast, if DNA molecules preferentially lie flat on the gate surface of the capacitive EIS sensor, a higher hybridization signal can be expected. Therefore, in addition to the ionic strength of the solution, the orientation of DNA molecules to the sensor surface has a strong impact on the expected DNA-hybridization signal [147,148,149]; the method of immobilization of probe ssDNA must be tailored correspondingly.

To achieve a high hybridization efficiency, the DNA hybridization is typically performed in a high-ionic strength solution, while the changes in the sensor signal induced by the DNA immobilization or hybridization are often read out in a low-ionic strength solution in order to reduce the Debye screening effect and, thus, to enhance the sensor performance [135,137,146]. A post-hybridization binding of intercalators or DNA binders to dsDNA molecules may be an effective way to distinguish the hybridization signal from an undesirable background noise caused by the nonspecific adsorption of target cDNA molecules. This way, a more accurate and reliable detection of the hybridization event with EIS structures can be achieved [146]. Since DNA binders react specifically with dsDNA, the changes in the sensor signal due to the binders could serve as an indicator to verify the successful hybridization process.

**Figure 5:**
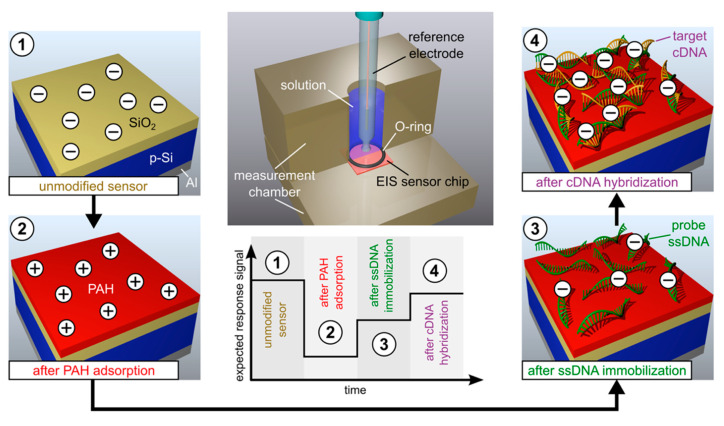
Modification steps of the EIS sensor, measurement setup (middle column, top), and expected ConCap response (middle column, bottom). (**1**) Unmodified sensor, (**2**) after poly(allylamine hydrochloride) (PAH) adsorption, (**3**) after single-stranded DNA (ssDNA) immobilization, and (**4**) after complementary target DNA (cDNA) hybridization. + and − symbols indicate the respective surface charges. Reproduced from Ref. [144] with permission of the American Chemical Society.

Besides the measurement of the DNA-hybridization signal in a low-ionic strength solution, reducing the distance between the DNA charge and sensor surface via the immobilization of DNA molecules flat to the EIS surface with a molecular charge lying within the Debye length from the gate surface is an essential factor to enhance the sensitivity of the DNA sensor. A direct adsorption of DNA molecules onto the EIS surface is, in general, hindered because of electrostatic repulsion forces between the negatively charged phosphate groups of the DNA and the negatively charged surface of the gate insulators typically used in EIS structures (e.g., SiO_2_ and Ta_2_O_5_). Therefore, an electrostatic adsorption of probe ssDNA molecules onto the EIS surface modified with a LBL-prepared positively charged PE layer and subsequent hybridization with cDNA molecules becomes more popular for designing DNA-EIS biosensors [130,132,137,141]. It is suggested that electrostatically adsorbed probe ssDNA molecules will be preferentially flat-oriented to the gate surface, with negatively charged phosphate groups directed to the cationic PE macromolecules, while the nucleobases will be exposed to the surrounding solution, allowing hybridization with the target cDNA molecules. In addition, due to the presence of a cationic PE layer, both the Debye screening effect and the electrostatic repulsion between probe ssDNA and target cDNA molecules will be less effective, resulting in an acceleration of the hybridization event, as well as a higher hybridization signal. Moreover, in contrast to frequently used, time-consuming, and cost-intensive covalent immobilization techniques, the LbL electrostatic adsorption method is easy, fast, and does not require complicated procedures for the functionalization of the gate surface and/or probe ssDNA molecules. For example, poly-L-lysine (PLL)-modified SiO_2_-gate EIS sensors were utilized for the detection of ssDNA immobilization and the DNA-hybridization process [130]. Although the sensor was able to detect low concentrations (2 nM) of target cDNA oligonucleotides (12-mer), the hybridization signal was small (several mVs). In further experiments, these EIS sensors were applied for monitoring PCR-amplified dsDNA [139,142]. Recently, in our group, the feasibility for the label-free electrical detection of DNA with capacitive SiO_2_-gate EIS sensors, which were modified with a positively charged weak PE of PAH, was demonstrated [132,137,141,144]. Figure 5 schematically shows the EIS-sensor surface before and after modification with PAH, probe ssDNA, and after cDNA hybridization; the measurement setup; and the expected ConCap response. High hybridization signals of 34 mV and 43 mV were recorded in low-ionic strength solutions of 10 mM and 1 mM, respectively [137]. In contrast, a small response of 4 mV was registered in the case of unspecific adsorption of fully mismatched DNA. These experiments demonstrated the specificity of the developed EIS sensor capable of distinguishing the complementary cDNA from fully mismatched DNA.

In a further work, PAH-modified EIS sensors were applied for the direct label-free electrical detection of dsDNA formed after a hybridization reaction occurred in the solution (so-called in-solution hybridization) for the first time [141]. Direct dsDNA detection could significantly simplify the surface modification procedure (because no probe ssDNA has to be immobilized onto the sensor surface) and, thus, may reduce the detection time and costs. Finally, the ability of PAH-modified EIS chips for the detection of PCR-amplified tuberculosis DNA fragments was demonstrated in [144]. The sensitivity of the sensor in artificial PCR solutions with different target cDNA (72-mer) concentrations from 1 nM to 5 μM was 7.2 mV/decade, with an estimated lower detection limit of ~0.3-nM cDNA. Such chips could serve as a sensing device for a quick verification of successful/unsuccessful DNA amplification by means of PCR.

Label-free field-effect DNA biosensors are typically disposable devices for a single-use measurement. To make DNA biosensors reusable, the complex surface/interface architecture should be regenerated, which is, in general, a complicated and time-consuming procedure [150]. On the other hand, it was reported that the surface of PE-modified DNA-EIS chips can be easily regenerated, making them suitable for multiple DNA immobilization and hybridization experiments [130,132,139]. As one example, the reusability of capacitive EIS sensors modified with PAH was examined for the detection of a ssDNA on-chip DNA hybridization event, as well as in-solution hybridized dsDNA molecules [132]. It has been demonstrated that the same biosensor can be reused for at least five DNA-detection measurements. For the experimental procedure, the simple regeneration of the gate surface of the EIS chip (covered with PAH/ssDNA or PAH/dsDNA layers) was realized by the electrostatic adsorption of a new positively charged PAH layer onto the negatively charged DNA layer. The performed experiment with the PAH-modified EIS sensors also allowed to investigate the impact of the Debye screening effect on the DNA immobilization and hybridization signal: the sensor response of the capacitive EIS sensor induced by the immobilization of ssDNA and dsDNA, as well as after the on-chip hybridization of cDNA, were recorded in solutions with different ionic strengths of 1 mM, 5 mM, 10 mM, and 20 mM. The evaluated Debye lengths amounted to approximately 9.6 nm, 4.3 nm, 3 nm, and 2.2 nm, respectively. The results of these experiments verified the assumption that, due to the more efficient screening of the DNA charge by counter ions, the amplitude of the DNA-immobilization and hybridization signal will decrease when increasing the ionic strength of the solution. For example, the on-chip cDNA-hybridization signal was reduced from 52 mV to 33 mV by increasing the ionic strength of the measuring solution from 1 mM to 20 mM [132].

The modeling of DNA-modified FEDs (DNA-FED), including DNA-EIS biosensors, is beneficial for understanding the mechanism of label-free DNA detection and for the optimization of device characteristics. Due to the high complexity of the DNA-modified gate insulator/electrolyte interface, to date, there are no exact theoretical models describing the functioning of DNA-FEDs taking into account all interfering factors. In addition to the counter-ion screening effect, other factors, like the orientation, length, and surface density of DNA molecules, charge distribution within the intermolecular spaces, distance between the DNA charge and the gate-insulator surface, gate surface-charge, etc., play a crucial role in converting the hybridization event to an electrical signal. Hence, several simplified theoretical models for DNA-FEDs were suggested and discussed. For example, a charge-plane model that takes into consideration both the Debye-screening length and the distance between the DNA charge and the gate surface was proposed in [135]. In another approach, in order to simulate the sensitive behavior of a DNA-FED, the DNA layer was modeled as an ion-permeable membrane [151]. The interplay between pH-, ion-, and charge sensitivity of FEDs modified with charged molecules was discussed in [152,153]. Moreover, the DNA hybridization-induced modulation of the ion-concentration distribution within the intermolecular spaces and ion sensitivity of the gate surface as a possible operation principle for DNA-FEDs was proposed in [154]. Recent simulations on the impact of the DNA position and orientation on the hybridization signal [149] show that the largest hybridization signal can be expected when the DNAs are parallel to the biosensor surface and distributed at equal intervals. Finally, the relation between the screening effect and the distance of the charged target molecule or particle from the electrolyte/insulator interface has been studied by using Monte Carlo simulation [155].

Summarizing this subsection, it should be emphasized that the majority of the reported label-free DNA-EIS biosensors utilize relatively short synthetic oligonucleotides as model targets and rather ideal experimental conditions. Problems may arise when dealing with real samples containing very large target DNA molecules (a thousand to several hundred thousands of base pairs) or other charged molecules (possible nonspecific adsorption).

#### 3.4.2. Detection of Biomarkers and Other Charged Molecules

Among the biomolecular interactions, the high specificity of molecular recognition can be best typified by an antibody–antigen interaction, which is the basis of immunosensors. Immunosensors as analytical devices have been recognized as very promising tools with enormous potential applications, including, e.g., monitoring contaminants in the environment, food safety, clinical diagnostics for monitoring the functioning of the immune system, etc. One of the key challenges of immunosensors is the detection of disease biomarkers that enable the early identification of diseases and effective treatments.

Label-free immunosensors allow the direct monitoring of immunoreactions by measuring physicochemical changes induced by the antigen–antibody complex formation. Immuno-sensitive FEDs (ImmunoFEDs) for label-free protein detection via their intrinsic molecular charge have attracted considerable interest due to their excellent sensitivity, fast response time, small size, cost-efficiency, and possibility of real-time and multiplexed measurements in a small sample volume and, therefore, are considered as promising alternatives to conventional immunoassays. ImmunoFEDs are often constructed by modification of the gate surface with antibodies as recognition elements for specific biomarkers (antigens). Since antibodies and antigens are generally electrically charged in aqueous solutions, it is suggested that the formation of an antibody–antigen complex on the gate surface will modulate the surface charge, inducing a biomarker concentration-dependent response of the FED. Most ImmunoFEDs reported in the literature are based on transistor structures (different types of ISFETs or SiNWs) [22,24], while capacitive EIS-based immunosensors (ImmunoEIS) have rarely been investigated. We only found a few studies related to the label-free direct detection of protein biomarkers and other molecules by their intrinsic charge with ImmunoEIS biosensors. Some examples of successful developments are described below.

A SiO_2_-gate EIS structure with APTES (3-aminopropyltriethoxysilane)-functionalized vertically aligned ZnO nanorods was utilized for the label-free detection of the prostate-specific antigen (PSA)—a biomarker strictly associated with prostate cancer [156]. The anti-PSA antibodies were covalently immobilized on the ZnO nanorods. Upon the binding of PSA of a concentration of 1 ng/mL in a 100-µM solution, a ~23-mV shift of the *C*–*V* curve was observed. In contrast, no significant voltage shift in the *C*–*V* curve was detected by measurements in a solution with an ionic strength of 10 mM, which was attributed to the counter-ion screening effect of the PSA charge. In a further work, PSA antigens were immobilized onto a polyethyleneimine-modified SiO_2_-gate surface, resulting in enhanced sensitive properties [131]. The sensitivity toward PSA molecules in a low-ionic strength solution was 28.2 mV/dec and 4.7 mV/dec in the PSA concentration range of 1–10 ng/mL and 10 pg/mL–1 ng/mL, respectively. The YbY_x_O_y_-gate EIS device was investigated for the detection of the rheumatoid factor (RF)—a diagnostic biomarker for rheumatoid arthritis [157]. RF antibodies functionalized with N-hydroxysuccinimide were covalently immobilized on the APTES-modified YbTi_x_O_y_ surface. The sensitivity of the biosensor to serum RF antigen was ~41 mV/dec in the concentration range of 0.1 µM–1 mM. Chand et al. used specific aptamers (instead of large antibodies) immobilized onto AuNPs deposited on the surface of a SiO_2_-gate EIS sensor for the label-free detection of protein kinase A (PKA) [158,159]. Wang et al. demonstrated the possibility of detecting bovine serum albumin (BSA) with HfO_2_-gate EIS sensors [107]. To avoid the complicated silanization process often used for antibody immobilization, the anti-BSA antibodies were immobilized on the HfO_2_ surface post-treated with NH_3_ plasma. The observed shift of the *C*–*V* curve along the voltage axis after the binding of BSA to anti-BSA was about 20 mV. An EIS sensor for direct monitoring of the binding of heparin molecules via detecting their intrinsic negative charge was proposed in [160]. Heparin is well-known as an important clinical anticoagulant, where low-molecular weight heparin is used for the prophylaxis of deep venous thromboembolisms. To prevent thrombosis and avoid bleeding risks during and after surgery, monitoring and control of the heparin level in a patient’s blood is important. The clinical heparin antagonist protamine or the physiological partner antithrombin III were used as heparin-specific surface probes. For the sensor, a detection limit of 0.001 U/mL was described [160], calculated from the dose-response curves; the achieved detection limit is in orders of magnitude lower than the clinically relevant concentrations. Ultimately, APTES-silanized Si_3_N_4_-gate capacitive EIS structures modified with magnetic nanoparticles were applied for ochratoxin A detection [161]. Ochratoxin A is one of the predominant contaminating mycotoxins in many products (e.g., dried fruits, coffee beans, beer, wine, etc.). The anti-ochratoxin A antibodies were immobilized on magnetic nanoparticles by amide bonding. The biosensor was highly sensitive (10 mV/pM in the linear range of 2.5–50 pM), with a detection limit of 4.57 pM and specific for ochratoxin A antigens, when compared to other interferences, such as ochratoxin B and aflatoxin G1.

In spite of the above-described successful experiments with ImmunoEIS sensors, detecting proteins (including disease biomarkers) and other charged molecules in real biological samples (e.g., whole blood, serum, or urine) remains a big challenge. The reasons are limitations in conjunction with the electrostatic detection of molecular charges with FEDs. The major limitation of the label-free electrostatic detection of proteins with ImmunoFEDs is the counter-ion screening of the molecular charge, already discussed in Section 3.4.1 for DNA-EIS sensors. The dimensions of some proteins (e.g., antibodies) are much larger (ca. 10–12 nm [162]) than the Debye length (~0.8 nm) in a solution (e.g., in whole blood), with an ionic strength of ~0.15 M. If immobilized antibodies are oriented such that the Fc (fragment crystallizable region) is substrate-facing (so-called end-on orientation), the distance between the binding sites and the sensor surface will be substantially greater than the Debye length. Thus, the target molecule/receptor binding will occur beyond the Debye length, making the electrostatic detection of the biomolecular charge in real samples difficult or even impossible. As a consequence, a useful measurable effect with an ImmunoEIS sensor can be expected only in low-ionic strength solutions (<10 mM). On the other hand, randomly adsorbed antibodies may have head-on, side-on, or lying-on orientations relative to the surface [163,164]. As a result, some of the bound target analyte charges can be expected to be held within the Debye length and, therefore, could be detectable by the ImmunoEIS sensor. Several strategies (e.g., measurements in desalted/filtered samples or in a low-ionic strength solution and the use of short receptors (aptamers and antigen-binding fragments)) have been proposed to reduce the influence of the counter-ion screening effect and, thus, to enhance the sensitivity of ImmunoFEDs (see reviews [22,23,153,165]).

Another issue is the nonspecific adsorption of proteins. Biological samples represent extremely complex media containing thousands of proteins and other charged chemical species (covering a very wide concentration range from pg/mL to mg/mL). They are able to nonspecifically adsorb on the gate surface of the ImmunoFED and generate false-positive signals or mask the usable signal from the target analyte of interest. This significantly hampers the sensitivity, specificity, and reliability of ImmunoFEDs and provokes false diagnostic results. To reduce/eliminate the nonspecific adsorption of proteins onto the surface of ImmunoFEDs, various strategies such as the use of blocking agents, prefiltering/purifying the biological liquids or on-chip filtering, separation, desalting, and preconcentration platforms, have been discussed for ImmunoFEDs [5,22,166].

#### 3.4.3. Detection of the Consecutive Adsorption of Oppositely Charged PE Macromolecules

Besides the detection of charged biomolecules such as DNA or proteins discussed above, EIS sensors have also been applied for the label-free detection of LbL sequential electrostatic adsorption of cationic and anionic PE molecules and the monitoring of a PE multilayer build-up. The LbL deposition of PE multilayers provides a simple and cost-effective method for the preparation of ultra-thin films (even organic/inorganic hybrid multilayers) with a desired composition, functionality, and nanoscale control of the thickness [167]. Such PE multilayers are very attractive as coatings with functional and controllable properties, stimuli-responsive materials for actuators, microcontainer-controlled drug release systems, and biosensing applications. For the optimization and practical implementation of PE multilayer-based devices, investigation of the impact of process parameters (e.g., PE concentration, ionic strength, pH value of the solution, and surface charge of the substrate) on the formation and characteristics of the PE multilayer is essential.

**Figure 6:**
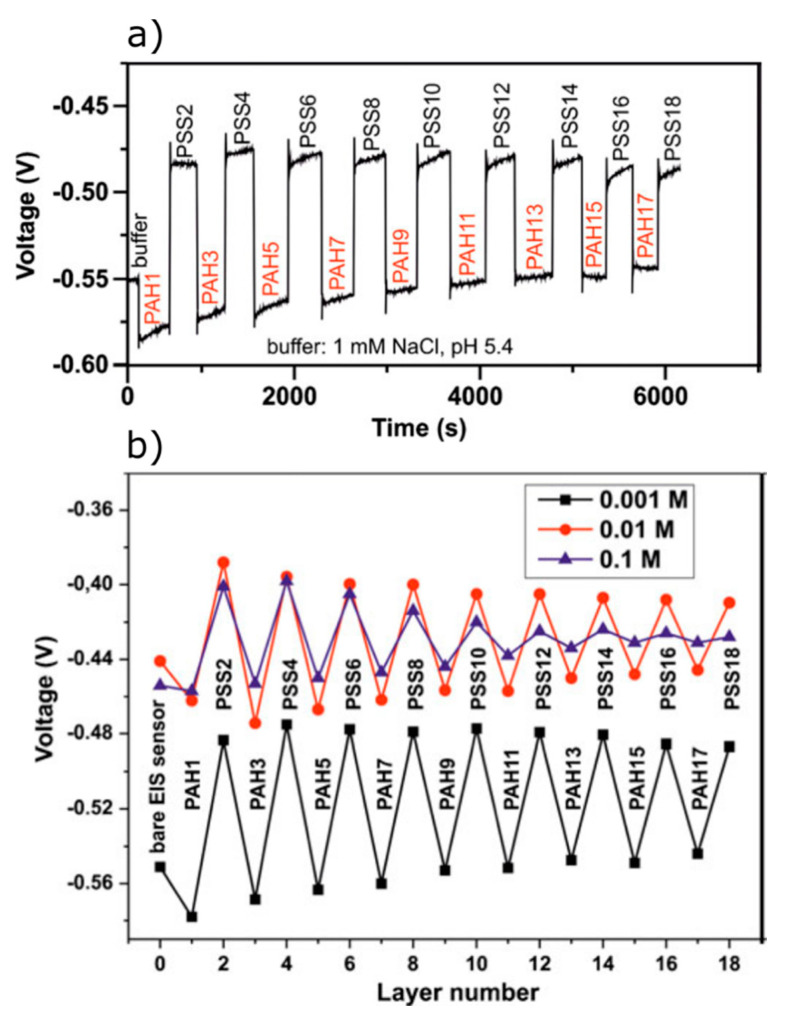
Electrical monitoring of the PE multilayer build-up with a p-Si–SiO_2_ EIS sensor. (**a**) ConCap response and (**b**) potential shifts as a function of the PE layer number and ion concentration. The PAH and poly(sodium 4-styrenesulfonate) (PSS) layers were deposited from a 50 μM PE solution adjusted with different NaCl concentrations of 100, 10, and 1 mM (pH 5.4). Reproduced from Ref. [172] with permission from Springer.

Recent studies on the detection of PE macromolecules using EIS structures with different gate materials (SiO_2_, SiO_2_–Ta_2_O_5_, SiO_2_–NCD, and SiO_2_–AuNP) and various PE model systems (PLL-ssDNA, PAH-PSS, PAH-ssDNA, and PAH-dsDNA) have demonstrated the potential of these EIS sensors for real-time, in-situ monitoring of the PE multilayer formation with direct electrical readout [7,8,30,130,132,168,169,170,171,172]. For example, the effect of the semiconductor doping type on the electrical characteristics of a PAH-ssDNA-modified SiO_2_-gate EIS structure was studied in [8]. The pH and ion sensitivity of a SiO_2_-gate EIS sensor covered with a PE multilayer of PAH-PSS was investigated in [170]. An array of nanoplate capacitive EIS structures prepared from a silicon-on-insulator wafer was applied for the electrical monitoring of the PE multilayer formation using differential-mode dynamic ConCap measurements [7]. The feasibility of an AuNP-modified capacitive EIS sensor for the label-free detection of the consecutive adsorption of cationic weak PE PAH and anionic strong PE PSS was demonstrated in [30]. More recently, the formation of a PAH-ssDNA and PAH-dsDNA multilayer onto SiO_2_-gate EIS sensors was studied in [132]. Finally, in the work of Poghossian et al., a PE multilayer stack (18 layers) of PAH-PSS onto a SiO_2_-gate EIS sensor was investigated in detail to understand the effect of ionic strength of the solution, PE concentration, and number and polarity of PE layers on the sensor signal [172]. Consecutive adsorption of oppositely charged PE layers leads to alternating shifts of the sensor signal of the capacitive EIS sensor, whereas the direction of these shifts correlates with the charge sign of the terminating PE layer (PAH or PSS). To interpret it in more detail: adsorption of a positively charged PAH layer shifts the signal of the capacitive EIS sensor in the direction for an additional positive charging of the gate surface. This corresponds to a more negative sensor output signal in the ConCap mode due to the feedback-control circuit (see Figure 6a). In contrast, PSS layer adsorption shifts the potential towards the direction that results from a more negatively charged gate surface. Subsequent PAH and PSS layers adsorption show a zigzag-like behavior in potential shifts (Figure 6b) that can be explained by the charge sign of the outermost layer. Similar effects were also found for PAH-PSS and PAH-DNA multilayers elsewhere [7,8,130,132,168,169,170,171]. In addition, it has been observed that the amplitude of the signal changes has a tendency to decrease with increasing the PE layer number and ionic strength (Figure 6b). To explain the experimentally observed signal behavior of an EIS sensor modified with a PE multilayer, a simplified electrostatic model was proposed, which is based on the assumption of a reduced ionic strength and, therefore, reduced screening of PE charges by mobile ions inside the multilayer [172]. According to this model, with increasing the multilayer thickness, the electrostatic coupling between the charge of the outermost PE layer and the gate surface will drop. As a result, the potential changes generated on the gate surface by the adsorption of the outermost PE layer will gradually decrease with an increasing number of PE layers that, in fact, was observed in many experiments. However, the development of exact theoretical models quantitatively describing the influence of all interfering factors on the signal behavior of PE-modified EIS sensors still requires further experimental input.

#### 3.4.4. Label-Free Biosensing with AuNP-Modified EIS Structures

Due to their unique physicochemical features, easy surface modifications with various shell molecules capable for coupling of different biochemical recognition elements, high surface-to-volume ratios, and the possibility of integration with macroscopic transducers, AuNPs are considered as highly attractive chemically and electrically tunable nanomaterials for designing electrochemical biosensors. In comparison with planar surfaces, the immobilization of receptors on nanoparticles typically provides a higher density of receptor molecules with a favorable orientation for interactions with target molecules to be detected, enhancing the transport of target molecules to the nanoparticle surface, all improving the biosensor performance. In this context, the coupling of AuNPs with FEDs represents a very promising strategy with new opportunities for label-free biosensing with direct electrical readout [30]. Since the vast majority of biomolecules are positively/negatively charged in solutions, AuNP-modified FEDs can provide a generic approach for detecting numerous charged biomolecules. Nevertheless, in spite of the popularity of AuNPs in electrochemical sensing, there are only a few articles devoted to the label-free detection of charged molecules with AuNP-modified capacitive EIS sensors [7,30,113,135,158,159].

As one example, a capacitive EIS sensor with SiO_2_ gate was applied to detect charge changes of ligand-stabilized and bare-supported AuNPs, which were induced by oxygen plasma treatment or by exposure to aqueous oxidation and reduction solutions, respectively [133]. Another, more recent experiment described capacitive EIS sensors functionalized with negatively charged, citrate-capped AuNPs for the label-free electrostatic detection of positively charged molecules by their intrinsic molecular charge [30,113]: charge changes can be detected in those AuNP/molecule inorganic/organic nanohybrids that result from molecular adsorption or binding events. Here, the ligand-stabilized AuNPs play a dual role: (i) The AuNPs offer a simple way to couple a large variety of charged molecules on their surfaces (e.g., negatively charged citrate-capped AuNPs provide a convenient scaffold to attach positively charged molecules), and (ii) the AuNPs can serve as additional distributed, quasi-spherical nanometer-sized, local metal gates [30].

**Figure 7:**
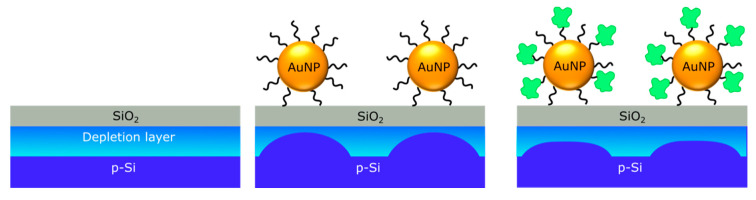
Schematic of a AuNP-modified capacitive EIS sensor. Expected modulation of the depletion layer after the deposition of negatively charged citrate-capped AuNPs and after the binding of positively charged molecules on AuNPs are exemplarily shown for a p-type EIS structure.

The expected modulation of the depletion layer in Si within local regions under surface areas covered with AuNPs is depicted in Figure 7: the adsorption or binding of charged molecules onto the AuNP surface will locally alter the width of the depletion layer and, therefore, the depletion capacitance. The overall capacitance of the field-effect EIS sensor will change, shifting the *C*–*V* curve along the voltage axis. The effect of coupling of charged molecules to the AuNPs is the same as if one would apply an additional voltage to the local gates. In which direction the voltage is shifting depends on the sign of the charge of the attached molecules, while the amplitude is determined by the surface density of AuNPs, the number of attached molecules per AuNP, and their intrinsic charge. Summarizing, a high sensor signal can be expected by a high AuNP surface coverage, a large number of highly charged, attached molecules per AuNP, and when performing measurements, in low-ionic strength solutions.

The feasibility of the proposed detection scheme has been exemplarily demonstrated by developing SiO_2_-gate EIS sensors modified with negatively charged citrate-capped AuNPs (with an average size of ~18 nm) for the detection of typical model examples of positively charged small proteins (cytochrome c, which is a key component of the electron transport chain in the mitochondria) and macromolecules (poly-D-lysine), as well as for monitoring the consecutive adsorption of oppositely charged PE molecules [30].

The possibility of detecting protein kinase A (PKA) with an AuNP-modified EIS structure was demonstrated in [158]. PKA has several functions in the cell, including the regulation of glycogen-, sugar-, and lipid metabolisms. AuNPs (~16 nm) functionalized with a thiolated PKA-specific aptamer were deposited onto the silanized SiO_2_-gate surface. The quantitative detection of PKA was performed by analyzing the *C*–*V* curve after the aptamer–PKA interaction. The EIS device showed a detection limit of 1-U/mL PKA. In a further work, the developed EIS sensor with an Ag/AgCl quasi-reference electrode was integrated with a polymeric microchip and tested for the label-free detection of PKA in a spiked human cell sample [159].

**Figure 8:**
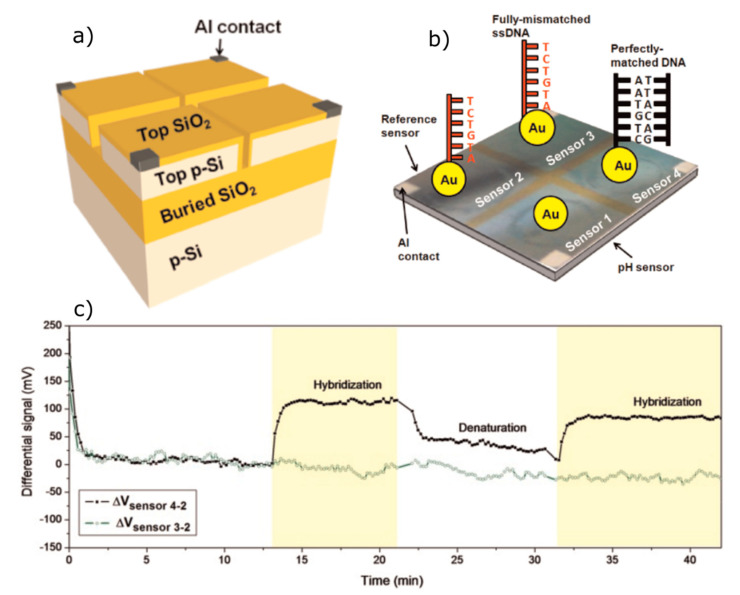
Schematic of the bare (**a**) and AuNP-modified (5–8 nm) (**b**) EIS chips combining four individually addressable EIS sensors prepared using a silicon-on-insulator wafer and a differential-mode ConCap response after consecutive DNA hybridization, denaturation, and rehybridization (**c**). ΔV_4-2_ and ΔV_3-2_: net differential signals between sensor 4 and sensor 2 and sensor 3 and sensor 2, respectively. Adapted from Ref. [135] with permission from Wiley-VCH.

Finally, an array of nanoplate EIS sensors functionalized with AuNPs was applied for the label-free detection of consecutive DNA hybridization, denaturation, and rehybridization in a differential-mode setup [7,135]. Figure 8 shows a schematic of the bare (a) and AuNP-modified (5–8 nm) (b) EIS chips, combining four individually addressable EIS sensors prepared using a silicon-on-insulator wafer and a differential-mode ConCap response after consecutive DNA hybridization, denaturation, and rehybridization (c). Sensor 4 was functionalized with the probe ssDNA (20-mer) molecules perfectly matched to the complementary target cDNA sequence, while sensors 2 and 3 were immobilized with the fully mismatched ssDNA. Sensor 1 was utilized for the pH control. High differential signals of about 120 mV, 90 mV, and 80 mV were observed between sensor 4 and sensor 2 after the DNA hybridization, denaturation, and rehybridization events, respectively. The observed hybridization signal was three to four times higher than those previously reported for SiO_2_-gate EIS sensors without AuNPs (24–33 mV) in [27,169].

## 4. Concluding Remarks

In spite of remarkable progress in the research and development of EIS sensors and the implementation of new strategies and ideas to improve the sensitivity characteristics in the last few years, it should be noted that there are still some issues that must be overcome before the commercialization of EIS biochemical sensors and their transfer from scientific labs to real life and widespread applications will appear. Some challenges related to ion-sensitive EIS, EnEIS, DNA-EIS, and ImmunoEIS sensors, and possible strategies proposed for their solutions, are discussed in the respective sections. As for other kinds of FEDs, due to the Debye screening effect and nonspecific adsorption, the label-free detection of charged biomolecules with EIS biosensors in untreated, real biological samples (e.g., whole blood, serum, plasma, saliva, urine, cerebrospinal fluid, and nasopharyngeal swab) with high sensitivity and specificity is still demanding.

From the application point of view, the development of an array of capacitive EIS sensors for multiplexed detection, as well as the realization of a stable, reliable, miniaturized integrated reference electrode, are two further tasks to be solved. For a correct functioning of field-effect EIS sensors, the reference electrode should provide a stable potential during measurements independent of the pH value of the solution or concentration of the dissolved species. This is usually achieved by applying the liquid-junction reference electrode (e.g., Ag/AgCl electrode), which is still bulky and often fragile and, therefore, limits seriously the large-scale application of EIS sensors. In order to achieve the full advantage of EIS sensors, a comparable small reference electrode must be realized. In this context, the development of miniaturized integrated solid-state reference electrodes compatible with Si technology is of great interest. Due to the difficulties in the miniaturization and integration of liquid-junction reference electrodes, some groups use so-called pseudo- or quasi-reference solid-state electrodes made from, for example, Pt, Au, or Ag/AgCl films. Such electrodes are often unsuitable for reliable biosensing because of non-negligible potential instabilities and the need of a long time to achieve a relatively stable potential after changing the electrolyte solution. Therefore, the results of experiments performed using quasi-reference electrodes should be carefully evaluated.

An ability of sensors for multiplexed detection—that is, to simultaneously assay for multiple chemical or biological species—could reduce both the analytical time (and cost) and sample volume. However, the integration of capacitive EIS sensors in an array format for multiparameter detection must circumvent technological difficulties in fabricating separate, individual, electrically isolated, field-effect capacitors onto the same Si chip [7]. Several capacitive EIS sensors fabricated on the same Si chip will stay interconnected through their common Si substrate. As a consequence, this can result in undesired cross-talk between the different EIS sensors on the same chip. Individually addressable on-chip fabricated EIS capacitors for multiparameter detection still remains a challenge, for which only a few studies have presented encouraging results [7,8]. The price to be paid is the loss of important advantages of capacitive EIS sensors—namely, the simple structure and easy preparation.

One widely neglected subject in most reported EIS sensors is the possible leakage current between the reference electrode and the Si substrate (ideally, no leakage current should flow). An existence of a leakage current might lead to serious experimental artifacts and even electrolysis, depending on the electrolyte composition and potentials applied to the system [173]. In spite of this fact, there are only a few works where data on leakage current levels in EIS sensors were given.

In summary, despite the above discussed issues, the research of capacitive field-effect EIS sensors is a rapidly advancing field in which novel device designs and modification methodologies are consistently being developed. This fact provides the reason for great optimism that capacitive EIS structures will play a significant role in the commercialization of FED-based chemical sensors and biosensors in the near future.

## Figures and Tables

**Table 1:** Characteristics of electrolyte-insulator-semiconductor (EIS) pH sensors with different pH-sensitive materials and deposition techniques.

pH-Sensitive Material	Deposition Method	pH Sensitivity, mV/pH	pH Range	Drift, mV/h	Hysteresis, mV	Reference
SiO_2_	LPCVD	41.5	2–10	19.6	19.4	[31]
SiO_2_ structured	LPCVD	52	2–10	1	11	[31]
SiO_2_ textured with SiO_2_ particles	TO of Si	43–54	4–10	16–40	5–6	[9]
SiO_2_	TO of Si	35–38	3–9	-	-	[30]
Si_3_N_4_	LPCVD	50	2–12	6	-	[57]
Si_3_N_4_	LPCVD	50	3–12	4	21	[32]
Al_2_O_3_	ALD	55	3–12	5.5	-	[33]
Al_2_O_3_	ALD	54.5	3–12	2	14	[32]
Al_2_O_3_	PLD	56	2–12	<1	3	[34]
Ta_2_O_5_	TO of Ta	57 ± 1.5	3–10	0.5	4	[29]
Ta_2_O_5_	TO of Ta	56	1–10	-	5	[35]
ZrO_2_	TO of Zr	50.6	2–10	-	-	[37]
HfO_2_	ALD	59.6	2–12	1	4.3	[41]
HfO_2_	RFS	51	2–10	1	25	[40]
HfO_2_	RFS	58.3	2–12	0.65	1.7	[39]
CeO_2_	RFS	58.8	2–12	1	6	[42]
Gd_2_O_3_	TO of Gd	53	2–10	5.4	-	[43]
Gd_2_O_3_	RFS	55	2–10	1.2	-	[44]
Ti-doped Gd_2_O_3_	RFS	55	2–12	1.4	3.6	[45]
Lu_2_O_3_	RFS	56	2–12	1.3	2.2	[46]
Nd_2_O_3_	RFS	56	2–12	1.3	4.7	[47]
Yb_2_O_3_	RFS	55.5	2–12	1.5	3.8	[48]
BST	sputtering	48–56	2–10	-	-	[54]
BST	PLD	57.4	3–11	-	2	[56]
NCD	MPECVD	54–57	4–11	-	-	[58]
Dy_2_TiO_5_	co-sputtering Dy/Ti	57.6	2–12	0.4	0.2	[49]
Er_2_TiO_5_	co-sputtering Er/Ti	58.4	2–12	1.2	4.6	[50]
PbTiO_3_	sol-gel	56–59	2–12			[51]
YTi_x_O_y_	sol-gel	58.5	2–12	0.1	2.6	[52]
Tm_2_Ti_2_O_7_	co-sputtering Tm/Ti	59.4	2–12	2.4	0.6	[53]

BST: barium strontium titanate, NCD: nanocrystalline diamond, LPCVD: low-pressure chemical vapor deposition, RFS: radio frequency sputtering, TO: thermal oxidation, ALD: atomic layer deposition, PLD: Pulsed laser deposition, and MPECVD: microwave plasma-enhanced chemical vapor deposition.

**Table 2:** Typical characteristics of enzyme-modified capacitive EIS (EnEIS) biosensors.

Analyte/Enzyme	pH Layer	Immobilization	Sensitivity	Detection Range, mM	LDL, µM	Ref.
acetoin/AR	Ta_2_O_5_	crosslinking	65 mV/dec	0.01–0.1	-	[80]
creatinine/creatinine deaminase	Dy_2_TiO_5_	covalent on magnetic bead	22–29 mV/dec	0.01–10	-	[81]
creatinine/creatinine deaminase	Dy_2_TiO_5_	entrapment in alginate bead	105 mV/dec	0.01–10	1	[82]
creatinine/creatinine deaminase	Tm_2_Ti_2_O_7_	entrapment in alginate gel	82 mV/dec	0.01–15	-	[83]
cyanide/cyanidase	Ta_2_O_5_	covalent	4 mV/dec	0.001–10	-	[84]
formaldehyde/FDH	Si_3_N_4_	entrapment	31 mV/dec	0.01–20	10	[85]
glucose/GOx	Dy_2_TiO_5_	entrapment in alginate bead	12 mV/mM	2–8	62	[82]
glucose/GOx	Ta_2_O_5_	LbL, PAH/ZnO/CNT/GOx	12 mV/dec	0.5–20	-	[92]
glucose/GOx	ZnO	crosslinking	3.1 V/mM	2–7	-	[89]
glucose/GOx	Tm_2_Ti_2_O_7_	encapsulation within hydrogel	14.7 mV/mM	2–8	-	[53]
glucose/GOx	Mg/ZnO	crosslinking	10.7 mV/mM	2–7	-	[95]
paraoxon/OPH	Ta_2_O_5_	crosslinking	~1 mV/µM	0.002–0.05	2	[96]
penicillin/PEN	Ta_2_O_5_	LbL, PAMAM/CNT/PEN	100 mV/dec	0.025–25	25	[94]
penicillin/PEN	NCD	adsorptive	85 mV/dec	0.005–2.5	5	[58]
penicillin/PEN	SiO_2_	LbL, PAH/PENe	100 mV//dec	0.025–10	20	[101]
Penicillin/PEN	Ta_2_O_5_	adsorptive	46 mV/dec	0.05–10	50	[102]
penicillin/PEN	Ta_2_O_5_	TMV nanocarrier	92 mV/dec	0.1–10	50	[103]
urea/urease	SiO_2_	LbL, Fe_3_O_4_-NP/PE/urease	32 mV/dec	0.1–100	100	[104]
urea/urease	Mg/ZnO	crosslinking	8.4 mV/mM	2–32	-	[95]
urea/urease	Dy_2_TiO_5_	entrapment in alginate gel	118 mV/dec	1–32	-	[105]
urea/urease	Ta_2_O_5_	LbL PAMAM/CNT/urease/CNT	33 mV/dec	0.1–100	-	[106]
urea/urease	HfO_2_	crosslinking	117 mV/dec	0.1–10	-	[107]

NCD: nanocrystalline diamond, dec: decade, LbL: layer-by-layer, PAH: poly(allylamine hydrochloride), PAMAM: polyamidoamine dendrimer, CNT: carbon nanotube, GOx: glucose oxidase, AR: acetoin reductase, OPH: organophosphorus hydrolase, FDH: formaldehyde dehydrogenase, PEN: penicillinase, NP: nanoparticle, PE: polyelectrolyte, TMV: tobacco mosaic virus, and LDL: lower detection limit.
